# Safety and efficacy of roux-en-y gastric bypass in older aged patients

**DOI:** 10.1590/0100-6991e-20223332-en

**Published:** 2022-09-21

**Authors:** JORGE HUMBERTO RODRIGUEZ QUINTERO, RACHEL GROSSER, GUSTAVO ROMERO VELEZ, VICENTE OMAR RAMOS-SANTILLAN, XAVIER PEREIRA, FERNANDO MUÑOZ FLORES, JENNY CHOI, ERIN MORAN-ATKIN, DIEGO CAMACHO, DIEGO LAURENTINO LIMA

**Affiliations:** 1 - Montefiore Medical Center, Surgery - The Bronx - New York - Estados Unidos

**Keywords:** Gastric Bypass, Bariatric Surgery, Anastomosis, Roux-en-Y, Derivação Gástrica, Cirurgia Bariátrica, Anastomose em-Y de Roux

## Abstract

**Introduction:**

laparoscopic Roux-en-Y Gastric Bypass (LRYGB) has been a revolutionary intervention for weight loss with reduction of up to 60-70% of excess body weight. However, these outcomes are not as well validated at the extremes of age, where the safety of the intervention still has some caveats. The aim of this study is to assess the efficacy and safety of primary LRYGB among different age groups.

**Methods:**

the Metabolic and Bariatric Surgery Accreditation and Quality Improvement Program (MBSAQIP) database was queried for patients who underwent primary LRYGB from January 2014 to December 2017 at a single institution. Four groups were created and compared by dividing our sample by age quartiles. The primary outcome was percent excess weight loss (%EWL) at 1 year. Additional operative outcomes and complications were also compared across groups.

**Results:**

a total of 1013 patients underwent non-revisional LRYGB during the study period. Mean %EWL at one year was 55%. When compared between quartiles, there was a statistically significant difference in %EWL: 1^st^ 62%, 2^nd^ 57%, 3^rd^ 54% and 4^th^ 47% (p=0.010). The differences in the secondary outcomes between age groups did not demonstrate statistical significance.

**Conclusions:**

though patients in the fourth age quartile (range) did not demonstrate a statistically significant increase in adverse outcomes, they did lose less weight compared to other cohorts. The %EWL at one year after RYGB varied by age in our cohort. Goals after bariatric surgery should be individualized as weight loss is less robust with aging.

## RESUMO

**Introdução:**

o Bypass Gástrico Laparoscópico em Y de Roux (LRYGB) tem sido uma intervenção revolucionária para perda de peso com redução de até 60-70% do excesso de peso corporal. No entanto, esses resultados não são tão bem validados nos extremos de idade, onde a segurança da intervenção ainda possui algumas ressalvas. O objetivo deste estudo é avaliar a eficácia e segurança do LRYGB entre diferentes faixas etárias.

**Métodos:**

O banco de dados do Programa de Acreditação e Melhoria da Qualidade da Cirurgia Metabólica e Bariátrica (MBSAQIP) foi consultado para pacientes submetidos a LRYGB de janeiro de 2014 a dezembro de 2017 em uma única instituição. Quatro grupos foram criados e comparados dividindo a amostra por quartis de idade. O desfecho primário foi perda percentual de excesso de peso (%EWL) em 1 ano.

**Resultados:**

1013 pacientes foram submetidos a LRYGB durante o período do estudo. A média de %EWL em um ano foi de 55%. Quando comparados entre os quartis, houve diferença significante no %EWL: 1º 62%, 2º 57%, 3º 54%, e 4º 47% (p=0,010). As diferenças nos desfechos secundários entre as faixas etárias não demonstraram significância estatística.

**Conclusões:**

embora os pacientes no quarto quartil de idade não tenham demonstrado um aumento estatisticamente significativo nos resultados adversos, eles perderam menos peso em comparação com outras coortes. O %EWL um ano após RYGB variou de acordo com a idade em nossa coorte. Os objetivos após a cirurgia bariátrica devem ser individualizados, pois a perda de peso é menos robusta com o envelhecimento.

## INTRODUCTION

Laparoscopic Roux-en-Y Gastric Bypass (LRYGB) is the gold standard bariatric surgical procedure as it allows for a reduction of up to 60-70% of excess weight loss (%EWL) at 2 years after surgery^1^ ^,^ ^2^. However, the reproducibility of safety and efficacy of this intervention in the extremes of age has been controversial^3^ ^-^ ^5^. In older, frail individuals, the perioperative and short-term safety of the intervention has been called into question; while in adolescent and younger individuals, the long-term metabolic and developmental effects are often cited as areas of concern and not well understood^6^ ^-^ ^9^. 

Older patients present a challenge to the bariatric surgeon. They tend to have medical and surgical comorbidities beyond metabolic syndrome that make them less favorable candidates for an elective intervention with potentially profound physiologic implications. In particular, they often have evidence of the long-term effects of metabolic syndrome on cardiopulmonary fitness, which affect perioperative morbidity and mortality. However, for precisely this reason, there is mounting evidence suggesting that the prevalence of obesity is rising in the older population. As such, we expect to see an increased number of these patients seeking bariatric surgery^10^ ^,^ ^11^. Some groups have proposed performing less morbid operations in elderly patients in an attempt to reduce their surgical morbidity while offering the best long-term outcomes^8^. However, this topic remains controversial as other bariatric procedures, such as the laparoscopic sleeve gastrectomy, have yet been proven to be superior to the RYGB^12^ ^,^ ^13^. 

There is scarce data on bariatric outcomes related to a patient’s physiologic age; most of the available literature compares younger versus older patients based on a threshold, without considering age as a continuum and conclude that outcomes are comparable^14^ ^-^ ^17^. Contreras et al., who used an age threshold of 45 years old, suggested that younger patients had a higher %EBMIL at one year without exploring the etiology of this difference^5^. The aim of our study was to assess outcomes of primary LRYGB across more nuanced age groups in order to detect a difference in %EWL based on age. 

## METHODS

### Study Design

This is a retrospective analysis of a prospective cohort. We use the Metabolic and Bariatric Surgery Accreditation and Quality Improvement Program (MBSAQIP) database. We identified and included all patients older than 18 years old who underwent primary RYGB from January 2014 to December 2017 at an academic tertiary high volume bariatric center. This study was approved by our Institutional Review Board, following the Ethics committee and HIPAA compliant mechanisms. The study performed accordingly with the principles stablished on the declaration of Helsinki (1964). Informed consent was waived due to the retrospective nature of the study.

### Data Collection

Patient baseline characteristics included age, gender, diabetes mellitus, smoking status, ASA class, height, highest weight, highest body mass index (BMI), and BMI at the day of surgery. Surgical characteristics included surgical technique, and operative time.

The primary outcome was percentage excess weight loss at 1 year (%EWL at 1 year). Secondary outcomes measured included percentage excess weight loss at 30 days (%EWL at 30 days), superficial surgical site infection (SSI), deep SSI, organ and space SSI, pneumonia, myocardial infarction (MI), postop transfusion, deep venous thrombosis (DVT)/ pulmonary embolism (PE), admission to intensive care unit (ICU), disposition after discharge, number of readmissions, and number of reoperations 30-days after the index operation. 

Standard definitions for outcomes reporting were used as outlined by the American Society of Metabolic and Bariatric Surgeons (ASMBS) for body mass index (BMI), and percent excess weight loss (%EWL)^18^. 

### Surgical Technique

In our Institution 3 ASMBS certified surgeons performed all the bariatric procedures during the study period. All patients underwent a multidisciplinary evaluation prior to surgery. All cases were done laparoscopically with a 50-75cm biliopancreatic limb and a 120-150cm alimentary limb. The gastro jejunal anastomosis was performed in an antecolic, ante gastric configuration using 3 different surgical techniques (handsewn, circular and linear stapler) which have been demonstrated to have similar %EWL at 1 year^19^. 

### Statistical Analysis

A análise estatística foi realizada usando o prograStatistical analysis was performed using SPSS Statistics 27.0 (IBM Corp., Armonk, NY, USA). The patient´s age underwent analysis of normality, utilizing the Kolmogorov-Smirnov test allowing for the division of our sample in four age quartiles. All variables underwent analysis of normality to confirm their distribution. Categorical variables were expressed as frequencies and percentages while continuous variables with a normal distribution were expressed as mean +/- standard deviation (SD), and those with not normal distribution were expressed as medians and ranges. Patient characteristics, operative variables and postoperative outcomes were compared between age groups using Chi square test for categorical variables and One-Way ANOVA test for continuous variables with a normal distribution.

Additionally, a correlation analysis using Pearson’s test was done to identify variables that correlated with %EWL at one year in the eldest quartile; followed by a linear multiple regression model to find predictors of %EWL at 1 year. A p-value of <0.05 was considered statistically significant.

## RESULTS

**Figure 1:**
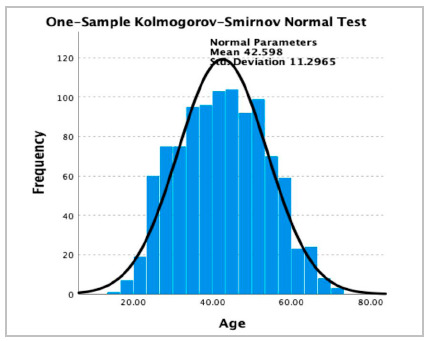
Histogram showing frequencies of different ages in our study population.

A total of 1013 patients underwent non-revisional LRYGB during the study period. Eighty five percent of our patients were female. The mean age in our sample was 42.5 years old (SD 11.29), and the cohort was divided into discreet age quartiles: 1^st^ (n=253), 2^nd^ (n=254), 3^rd^ (n=254) and 4^th^ (n=252) (Figure 1). The mean age for each one of the groups was 1^st^ 28.1 years old (SD: 3.86), 2^nd^ 38.3 years old (SD 2.48), 3^rd^ 46.8 years old (SD 2.56), and 4^th^ 57.3 years old (SD 4.66). 

The summary of the patient’s characteristics is shown in Table 1. Patients in the eldest age quartile (4^th^ group) demonstrated a higher prevalence of diabetes, 23% (n=58) of whom were insulin dependent and 29% insulin independent (n=73) (p<0.001), higher ASA class status (p<0.001), lower height (p<0.001), lower max weight (p<0.001), lower max BMI (p<0.001), and lower BMI on day of surgery (p<0.001).

**Table 1:** Características basais e grupos.

		1^st^ Quartile	2^nd^ Quartile	3^rd^ Quartile	4^th^ Quartile	p value
	coorte n=1013	n=253	n=254	n=254	n=252	
Age	42.5 (11.29)	28.09 (3.86)	38.27 (2.48)	46.80 (2.56)	57.26 (4.66)	NA
Gender						
Female	858(85%)	214 (85%)	216 (85%)	212 (84%)	216 (86%)	0.915
Male	155(15%)	39 (15%)	38 (15%)	42 (16%)	36 (14%)	
Diabetes						
IDDM	133 (13%)	12 (5%)	29 (11%)	34 (14%)	58 (23%)	<0.001
NIDDM	224 (22%)	31 (12%)	48 (19%)	72 (28%)	73 (29%)	
Smoker	85 (8%)	27 (11%)	21 (8%)	21 (8%)	16 (6%)	0.378
ASA						
2	276 (27.2%)	97 (38.3%)	94 (37%)	54 (22%)	31 (12.3%)	<0.001
3	733 (72.5%)	155 (61.3%)	160 (63%)	198 (78%)	220 (87.3%)	
4	3(0.3%)	1 (0.4%)	2 (0%)	1 (0%)	1 (0.4%)	
		1^st^ Quartile	2^nd^ Quartile	3^rd^ Quartile	4^th^ Quartile	p value
Highest BMI (kg/m^2^)	46.02 (6.39)	48.02 (6.91)	46 (6.18)	45.40 (6.14)	44.65 (5.81)	<0.001
BMI Day of surgery	44.84 (6.47)	46.90 (6.68)	44.89 (6.15)	44.31 (6.72)	43.24 (5.78)	<0.001
Technique						
Linear stapler	146 (14%)	26 (10%)	30 (11.8%)	37 (14.6%)	53 (21%)	0.002
Handsewn	434 (43%)	130 (51%)	103(40.6%)	103(40.6%)	98 (38.9%)	
Circular stapler	433 (43%)	97 (38%)	121(47.6%)	114(44.9%)	101(40.1%)	
Duration of Surgery (min)	108.57(29.83)	103.72(24.63)	108.96(28.62)	110.16(27.75)	111.44(36.62)	0.02

NA= not applicable; IDDM= insulin dependent diabetes mellitus; NIDDM= non-insulin dependent diabetes mellitus; ASA= American Society of Anesthesiology; BMI= body mass index. Bold p-values are statistically significant. *Variables are presented as frequencies are percentages for categorical and mean (standard deviation for continuous).

The duration of the surgery was significantly different between age groups, with the eldest group having the longest time (111 minutes, p=0.02). Three different techniques for the gastrojejunal anastomosis were utilized as previously mentioned: linear stapler (14%), handsewn (43%), and circular stapler (43%) with a statistically significant difference in techniques between groups (p=0.002). 

The mean %EWL at 1 year after surgery for the entire cohort was 55.1% with significant differences seen between the age groups. The oldest group had the lowest %EWL at 1 year of all groups (1^st^ 62.4%, 2^nd^ 57.2%, 3^rd^ 53.6% and 4^th^ 46.6% (p=0.010)). The %EWL at 30 days was not different between groups (1^st^ 14.3%, 2^nd^ 15.1%, 3^rd^ 14.8% and 4^th^ 14.8% (p=0.406)). There was no statistically significant difference between groups in the rest of the outcomes variables compared as shown in (Table 2).

**Table 2:** Primary and secondary outcomes based on age groups.

	All cohort	1^st^ Quartile	2^nd^ Quartile	3^rd^ Quartile	4^th^ Quartile	p-value
	n=1013	n=253	n=254	n=254	n=252	
Age		28.09±3.86	38.27±2.48	46.80±2.56	57.26±4.66	
%EWL at 1 year	55.16(48.28)	62.45(29.31)	57.20(35.82)	53.67(39.70)	46.65(5.52)	0.01
%EBWL at 30 days	14.80(5.67)	14.30(13.38)	15.13(5.58)	14.89(5.73)	14.87(6.15)	0.406
Superficial SSI	19(2%)	3(1%)	9(4%)	3(1%)	4(2%)	0.153
Deep SSI	1(0.1%)	0	1(0.4%)	3(1%)	0	0.393
Transfusion	22(2%)	2(1%)	9(4%)	6(2%)	5(2%)	0.27
DVT/PE	4(0.4%)	2(1%)	1(0.4%)	1(0.4%)	0	0.571
ICU	3 (0.3%)	0	2 (1%)	1 (0.4 %)	0	0.301
LOS (Days)	1.80(0.92)	1.71(0.77)	1.76(1.01)	1.83(1.03)	1.89(0.83)	0.148
Disposition						0.173
Home	1010(99.7%)	251(99%)	254(100%)	253(99.6%)	254(100%)	
SNF	2(0.2%)	2(1%)	0	1(0.4%)	0	
Number of readmissions						0.61
0	970(95.7%)	241(95.2%)	242(95.3%)	245(96.5%)	242(96%)	
1	40(4%)	10(4%)	12(4.7%)	8(3.1 %)	10(4%)	
2	3(0.3%)	2(0.8%)	0	2(0.4%)	0	
Number of reoperations						0.545
0	0(99.7%)	253(100%)	253(99.6%)	252(99.2%)	252(100%)	
1	2(0.2%)	0	1(0.4%)	1(0.4%)	0	
2	1(0.1%)	0	0	1(0.4%)	0	
%Follow up at 30 days	1012(99.9%)	253(100%)	254(100%)	253(99.6%)	252(100%)	0.393

%EWL at 1 year= percent excess weight loss at 1 year; %EWL at 30 days = percent excess weight loss at 30 days; SSI= Surgical Site Infection; NA= not applicable; DVT= deep venous thrombosis; PE= pulmonary embolism; ICU= intensive care unit; LOS= length of stay; SNF= skill nursing facility. Bold p-values are statistically significant. *Variables are presented as frequencies are percentages for categorical and mean (standard deviation for continuous).

In the oldest age group, BMI before surgery (p<0.001), highest weight (p<0.001), minimal weight at 30 days (p<0.001), height (p<0.001) and highest BMI (p=0.036) had a significant correlation with %EWL at 1 year (Table 3). Lastly, a linear regression model was done to find predictors of %EWL at 1 year in the oldest quartile of age. Only height was independently associated with %EWL at 1 year (p<0.001) after controlling for all other factors. These findings are shown on Table 4.

**Table 3:** Correlation analysis with %EWL at 1 year in 4 ^th^ quartile of Age.

Variable	Pearson	p-value
%EWL at 30 days	-0.08	0.235
Minimal Weight at 30 days	0.304	<0.001
LOS	-0.023	0.751
Operative time	0.005	0.946
BMI before surgery	0.39	<0.001
Highest BMI	0.152	0.036
Highest weight	0.306	<0.001
Height	0.291	<0.001

%EWL= percent excess weight loss; LOS= length of stay; BMI= body mass index. Bold p-values are statistically significant.

**Table 4:** Linear regression model predicting %EWL in the 4th quartile of age.

Variable	OR	95% CI	p-value
Technique	1.146	(-2.669 - 10.061)	0.253
Height	3.382	(3.424 - 13.028)	<0.001
Highest weight	-0.94	(-3.328 - 1.181)	0.348
Highest BMI	1.36	(-4.144 - 22.490)	0.176
BMI day of surgery	0.439	(-9.647 - 15.160)	0.661
Duration of surgery	0.148	(-0.124 - 0.144)	0.883
LOS	0.702	(-4.329 - 9.112)	0.483
Number of readmissions	-0.043	(-30.211 - 28.919)	0.966
Variable	OR	95% CI	p-value
Min weight at 30 days	-0.562	(-3.195 - 1.778)	0.575
%EWL at 30 days	-0.985	(-4.748 - 1.587)	0.326
%PEP em 30 dias	-0,985	(-4,748 - 1,587)	0,326

*OR= odds ratio; BMI= body mass index; LOS= length of stay; %EBWL= percent excess weight loss. Bold p-values are statistically significant.*

## DISCUSSION

The surgical treatment of obesity has been established as superior to intensive medical therapy in achieving optimal and sustained weight loss as well as offering superior amelioration of comorbidities^1^ ^,^ ^20^. It has also demonstrated to have an acceptable safety profile that certainly justifies performing an elective procedure on a patient with multiple comorbidities^2^. However, our collective understanding of the effect of chronologic age on the efficacy and safety of bariatric surgery is poorly understood, and the number of patients with advanced age seeking surgical treatment of obesity will continue to increase in the near future^10^ ^,^ ^11^. 

In our study, we demonstrated that %EWL at 1 year varies according to age groups and is significantly lower in our oldest age group. Furthermore, we show a statistical significance in weight loss between successive age groups, which delineates an inversely proportional relationship between aging and weight loss after surgery. Prior studies have conflicting evidence regarding the effect of age on weight loss^3^ ^,^ ^5^ ^,^ ^8^ ^,^ ^21^ ^-^ ^23^. As an example, St Peter et al., demonstrated decreased weight loss in patients >60 years old when analyzing a smaller cohort of 110 patients^23^. Whereas O’Keefe et al. found adequate weight loss on their group >65 years. However, they focused on describing the weight loss and postoperative outcomes of older patients without comparing with a control group^24^. To our knowledge our study is the first to focus on evenly distributed age groups rather than a single threshold to identify older patients which allowed us to develop a more nuanced understanding of the role of age on weight loss. Our findings suggest that chronologic age plays an important role in %EWL with progressively less weight loss with increasing age. 

This finding has several important clinical implications. Making this distinction can help us counsel patients in individualizing their weight loss goals as well as perform a clearer cost-benefit analysis in determining the utility of this surgery in an older patient with multiple comorbidities. 

Even if weight loss is less substantial in this population, it can still have a significant impact on the overall health and quality of life of older patients. Patients who undergo bariatric surgery have lower mortality at 5 years and have an improvement of the atherosclerotic cardiovascular disease risk^1^ ^,^ ^25^. In a recent study based on the Scandinavian Obesity Surgery Registry, patients above 60 years old were found to have improvement in their comorbidities despite being a lower improvement compared to younger patients6. The multi-factorial benefits of this intervention have been extensively demonstrated and certainly justify its implementation. Having a clear risk benefit discussion with our patients can help us make more informed clinical decisions. 

The etiology of this finding also warrants further exploration and can be hypothetically attributed to multiple factors. Older patients are believed to have impaired metabolic capacity, sarcopenia and a lower level of physical activity^5^ ^,^ ^8^ ^,^ ^26^. However, this has only been described in the setting of patients older than 65 years of age, and more studies are necessary to elucidate if these findings are present across a wider age range. Additionally, older patients tend to have more established dietary habits that may be more difficult to change. As such, they could have poor adherence to the bariatric specific diet and other nutritional protocols. Lastly the older population has a higher incidence of mood disorders such as major depression, and associated comorbidities that can influence their baseline health condition and capacity to follow protocols. Further research aimed to understanding the driving factors behind this finding will help us further optimize our patient outcomes and counsel them on how to gain the most from their bariatric intervention. 

In terms of safety, our study demonstrated that bariatric surgery in older patients poses no greater risk of perioperative morbidity compared to younger patients. The current literature has mixed findings on this topic. Two systematic reviews that compared outcomes between older and younger patients support our findings^26^ ^,^ ^27^. The studies that contrast include one that notes increased mortality in Medicare beneficiaries older than 75 and 65 years old^28^ ^,^ ^29^. and an analysis of the American College of Surgeons-National Surgical Quality Improvement Project (ACS-NSQIP) database on patients >70 years undergoing bariatric interventions which found increased mortality and morbidity in the older group^7^. However, all of the authors of these studies conclude that even though their studies showed increased incidence of complications and mortality, those rates are still acceptable enough to deem the intervention safe. 

On a secondary analysis looking for factors associated with %EWL at 1 year in the oldest group, only height was found to be significant. This is likely due to the mathematic effect that height has on the ideal BMI of 25 that is considered when calculating %EWL. At a constant weight, height is inversely proportional to the BMI and directly proportional to excess weight loss. Therefore, we do not believe that this facet has clinical significance. 

Our study has important limitations. As a retrospective analysis it can be prone to selection bias. Additionally, as the four groups are different in regard of their baseline characteristics, confounding can be present, however we anticipated this as older patients tend to have more comorbidities and, therefore, have a higher incidence of diabetes and a higher ASA class. 

Even though we found differences in %EWL at 1 year, we did not address if these differences prevail during longer follow-up. As these patients will continue to be followed by our institution and we anticipate further follow-up studies in the future. 

Some of the strengths of the study is the ample sample size compared to prior studies from a single institution. Furthermore, we included only patients that underwent primary RYGB and no other bariatric procedures which controls for the various unique complications of each type of weight loss surgery.

## CONCLUSION

In conclusion, there is an inverse correlation between increasing age an %EWL at one year after RYGB. Goals after bariatric surgery should be individualized as weight loss is less robust with aging. Perhaps, the decision to perform bariatric surgery and the measurement of success in older groups should be focused on their comorbidities and lifespan instead of BMI as part of a careful cost-benefit analysis of the utility of bariatric surgery in each individual patient. 
