# Erratum

**DOI:** 10.1590/0100-6991e-20213332ER

**Published:** 2022-11-23

The article “SAFETY AND EFFICACY OF ROUX-EN-Y GASTRIC BYPASS IN OLDER AGED PATIENTS”, DOI number: 10.1590/0100-6991e-20213332, published in the Journal of the Brazilian College of Surgeons. 49(1); e20213332.

Where it is: 

Quintero “Safety and Efficacy of Roux-en-Y Gastric Bypass in Older Aged Patients”.

It should be: 

Rodriguez-Quintero “Safety and Efficacy of Roux-en-Y Gastric Bypass in Older Aged Patients”.

